# Genetic and Phenotypic Spectrum of Amyotrophic Lateral Sclerosis Patients with *CCNF* Variants from a Large Chinese Cohort

**DOI:** 10.1007/s12035-023-03380-1

**Published:** 2023-05-12

**Authors:** Bi Zhao, Qirui Jiang, Junyu Lin, Qianqian Wei, Chunyu Li, Yanbing Hou, Bei Cao, Lingyu Zhang, Ruwei Ou, Kuncheng Liu, Tianmi Yang, Yi Xiao, Rui Huang, Huifang Shang

**Affiliations:** 1 Department of Neurology, Laboratory of Neurodegenerative Disorders, West China Hospital, Sichuan University, Chengdu, 610041 Sichuan China; 2 Department of Neurology, Sichuan Academy of Medical Science and Sichuan Provincial People’s Hospital, Chengdu, China

**Keywords:** Amyotrophic lateral sclerosis, *CCNF* gene variant, Clinical characteristics, Genotype

## Abstract

*Cyclin F (CCNF)* variants have been found to be associated with amyotrophic lateral sclerosis (ALS)/frontotemporal dementia (FTD). However, the genetic and clinical characteristics of ALS patients who carry *CCNF* variants are largely unknown. Genetic analysis was performed for 1587 Chinese ALS patients, and missense variants were predicted by software analyses. Additionally, we searched PubMed, Embase, and Web of Science for relevant literature and conducted a meta-analysis of the frequency of variants. In our ALS cohort, we identified 29 nonsynonymous variants in 41 ALS patients. Among these ALS patients, 18 (1.1%) were carriers of 15 rare missense variants that were considered probably pathogenic variants, and 11 of 15 variants were novel. Seven relevant studies were identified, and a total of 43 *CCNF* variants in 59 ALS patients with a frequency of 0.8% were reported. The ratio of males to females in our cohort (10/8) was similar to that in Caucasian populations (4/7) and significantly higher than that in Asian populations (10/1). The proportion of bulbar onset in Caucasian *CCNF* carriers was similar to our cohort (25.0 vs. 27.8%); however, bulbar onset had never been reported in previous Asian studies (0/11). FTD was not found in *CCNF* carriers in previous Asian studies and our cohort, but it has been reported in a FALS cohort (1/75) of Caucasian individuals. There were some differences in the clinical characteristics among different ethnic ALS populations. More basic scientific studies are needed to elucidate the pathogenic mechanisms and genotype-phenotype associations of *CCNF* variants.

**Supplementary Information:**

The online version contains supplementary material available at 10.1007/s12035-023-03380-1.

## Introduction

Amyotrophic lateral sclerosis (ALS) is a devastating and rapidly progressive neurodegenerative disease. There are clinical heterogeneities among different patients; these include heterogeneities in age of onset, initial symptoms, clinical subtypes and disease progression. Approximately 10–15% of ALS patients are also diagnosed with frontotemporal dementia (FTD), and particularly, these patients carry chromosome 9 open reading frame 72 gene *(C9orf72)* mutations [1, 2]. Most patients die from respiratory failure within 3–5 years after onset, and the number of ALS cases is estimated to grow to approximately 380,000 by 2040 due to an aging population [3]. However, the etiology of ALS is still poorly understood. Genetic studies have shown that more than 50 genes are involved in the pathogenesis of ALS, and approximately 70% of familial cases can be explained by known gene mutations [4].

Recently, a single *cyclin F* (*CCNF)* variant that segregated with seven affected family members was identified by whole-genome linkage analysis and whole-exome sequencing (WES) [5]. Furthermore, screening for *CCNF* variants in ALS/FTD cohorts from diverse geographic populations (mostly European ancestry) revealed that *CCNF* variants in different ethnic ALS cohorts and novel and rare protein-altering *CCNF* variants were significantly enriched in sporadic ALS (SALS) patients [5]. *CCNF* encodes cyclin F, which is a component of the E3 ubiquitin-protein ligase complex (SCF^cyclin F^) that mediates protein ubiquitination and proteasomal degradation [6]. Functional analysis using neuronal cells revealed that mutant cyclin F leads to the abnormal accumulation of ubiquitinated proteins, including TAR DNA-binding protein of 43 kDa (TDP-43), and dysfunction in protein homeostasis [5].

However, the genetic spectrum and clinical phenotypes of *CCNF* variants in ALS patients are not fully understood due to a lack of sufficient samples and studies. Therefore, to further understand the role of *CCNF* variants in ALS, we screened 1587 ALS patients from southwestern China using WES and investigated the clinical characteristics of the patients carrying *CCNF* variants.

## Materials and Methods

### Study Population and Assessment

A total of 1587 definite or probable ALS patients diagnosed according to El Escorial ALS criteria [7] were included in the current study, all of whom were from the Department of Neurology, West China Hospital, Sichuan University, China. Familial ALS (FALS) is defined as a patient's first-, second-, or third-degree relative with ALS. The rest of the cases are classified as SALS. This study was approved by the Ethics Committee of West China Hospital of Sichuan University, and all recruited patients signed an informed consent form before participating in the study.

Demographic and clinical information was collected for all patients, and the patients were followed up in person or by telephone every three or six months. The severity of disease was assessed by the ALS function rating scale-revised (ALSFRS-R) at baseline and visits. The disease progression rate at baseline was calculated as (48 - “baseline” ALSFRS-R score) / the time from onset to “baseline” (months), and the follow-up disease progression rate was calculated as (“baseline” ALSFRS-R score - “last follow-up” ALSFRS-R score) / the time between “last follow-up” and “baseline” (months). Rapid progression was defined when the disease progression rate was greater than 0.5 [8]. The frontal assessment battery (FAB) [9] was used to assess frontal lobe function. The Montreal Cognitive Assessment (MoCA) was used to evaluate global cognitive function [10]. In our study, impairment of the frontal lobe and cognitive function was defined as a score of FAB ≤ 14 and MoCA ≤ 22, respectively [11, 12]. The Hamilton Depression Scale (HAMD, 24 items) [13], Hamilton Anxiety Scale (HAMA) [14], and Beck Depression Scale (BDI) [15] were used to evaluate mood. When the HAMD/HAMA score was > 7 or the BDI score was ≥ 5, the patient was considered to have symptoms of anxiety or depression.

### Genetic Analysis

The genetic analysis of patients was performed using WES and repeat-primed polymerase chain reaction (RP-PCR), in which RP-PCR was used to detect G4C2 repeats in *C9orf72.* Genomic DNA was collected from peripheral blood leukocytes using standard phenol–chloroform procedures. We choose 41 known ALS-related genes based on our previous study [16]. Rare missense variants were defined as having a minor allele frequency (MAF) < 0.1% in the Exome Aggregation Consortium-East Asian (ExAC_EAS) and Genome Aggregation Database-East Asian (GnomAD_EAS). In addition, we found the RefSeq ID of the transcript sequence of each *CCNF* variant. The novel variant is defined as a variant that has not been reported in ALS patients in previous studies. We enrolled ALS patients with the *CCNF* variant, and excluded the ALS patients with ALS gene classified as definitive on the ALSoD website (https://alsod.ac.uk/).

Sorting Intolerant from Tolerant (SIFT), Polymorphism Phenotyping v2 (PPH2), Functional Analysis through Hidden Markov Models (FATHMM), Combined Annotation Dependent Depletion (CADD), Mutation Assessor (MA), Deleterious Annotations of genetic variants using Neural Networks (DANN), Protein Variation Effect Analyzer (PROVEAN), and Likelihood Ratio Test (LRT) were used to predict the harmfulness of *CCNF* variants. Genomic Evolutionary Rate Profiling++ (GERP++) was used to predict nucleic acid conservation. If more than four of the eight software programs predicted the variant to be deleterious (prediction ≥ 4/8), the variant was considered probably pathogenic (PP) in our study.

### Search of the Literature

We searched articles on *CCNF* variants in ALS patients using the keywords (“amyotrophic lateral sclerosis” or “ALS” or “motor neuron disease”) and (“CCNF” or “Cyclin F”) in PubMed, Embase, and Web of Science (from inception until June 2022).

### Statistical Analysis

Statistical analysis was performed using the statistical software SPSS (IBM SPSS Statistics for Windows, version 26, IBM Corporation, Inc., Chicago, IL, USA, 2019). Wilcoxon's *T* test or rank-sum test was used to compare the clinical characteristics of carriers of the *CCNF* missense variants. The Kaplan–Meier method was used for survival analysis. We performed a meta-analysis of variant frequency using STATA 16.0 software (version 16.0; Stata Corp., College Station, TX, USA) to calculate effect sizes and 95% confidence intervals (CIs) for each previously reported study.

## Results

### Genetic Analyses

**Fig. 1:**
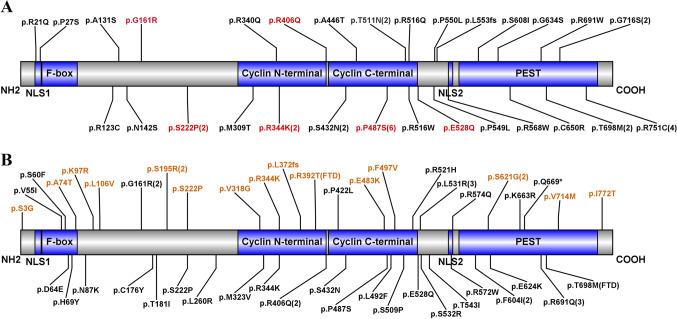
The distribution of *CCNF* variants at the protein level in our ALS cohort and previously reported cohorts

Among the 1587 recruited ALS patients, 29 nonsynonymous variants in the *CCNF* gene, including 28 rare missense variants and one frameshift variant, were identified in 41 ALS patients (Supplementary Table 1). Among the 29 variants, six variants (p.Gly161Arg, p.Arg344Lys, p.Arg406Gln, p.Glu528Gln, p.Pro487Ser, and p.Ser222Pro) have been reported in previous studies [5, 17–22]. Ten variants were located in the cyclin domains, seven variants were located in the PEST sequence, three variants were located in the nuclear localization signal (NLS), and nine variants were not located in any of the above domains (Fig. 1A). Using eight software programs to predict protein structure or function, 15 rare missense variants detected in 18 ALS patients were considered PP variants, of which 11 variants detected in 13 ALS patients were novel (Supplementary Table 2).

Interestingly, seven variants (p.Arg751Cys, p.Gly716Ser, p.Thr698Met, p.Arg691Trp, p.Cys650Arg, p.Gly634Ser, and p.Ser608Ile) located in the PEST sequence were all predicted to be nonpathogenic by software (prediction < 4/8) in our study.

### Demographics and Clinical Characteristics of *CCNF* Carriers

**Fig. 2:**
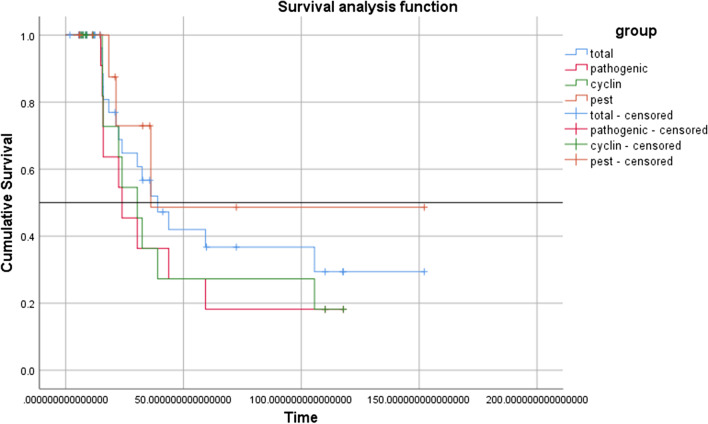
Survival analysis of ALS patients with *CCNF* variants in our cohort

The demographic and clinical characteristics of the patients with *CCNF* variants are shown in Table 1. In our cohort, no patients carrying *CCNF* variants had a positive family history. The mean age of onset of all patients with *CCNF* variants and those with PP variants were 57.13 ± 10.12 and 59.67 ± 8.79 years, respectively. Approximately one-third of patients carrying *CCNF* variants had bulbar onset. The proportion of *CCNF* variant carriers with a rapidly progressive disease rate decreased from baseline to the last visit (58.5 to 25.0%). At the last follow-up visit, 20 patients were lost to follow-up, five patients were still alive, and 16 patients died from respiratory failure or nutritional problems. The median survival times of all *CCNF* variant carriers and PP missense variant carriers were 39.00 ± 7.83 months and 23.97 ± 7.96 months, respectively (Fig. 2). There were no significant differences in the clinical characteristics between *CCNF* PP carriers and ALS patients who did not carry the *CCNF* gene variant and other identified pathogenic gene mutations. In addition, there were no significant differences in the clinical features, including nonmotor symptoms, between the two patient groups with variants in the cyclin domains and PEST sequence (Table 1).

**Table 1:** Demographic and clinical characteristics of ALS patients carrying *CCNF* gene variants in the study

Variables	All (*n*=41)	Pathogenic (*n*=18)	Cyclin (*n*=17)	Pest (*n*=12)	Control (*n*=90)	*p* value
Sex (F/M)	17/24	8/10	7/10	5/7	40/50	P1=1,P2=0.98
Years of education, n	34, 6.53 ± 4.00^a^	17, 5.47 ± 3.56^a^	14, 6.36 ± 5.37^a^	11, 7.55 ± 2.70^a^	6.77 ± 4.97^a^	P1=0.57,P2=0.51
BMI, kg/m^2^, n	33, 21.61 ± 2.99^a^	16, 22.95 ± 2.81^a^	14, 21.83 ± 2.41^a^	10, 20.55 ± 3.26^a^	21.78 ± 2.78^a^	P1=0.09,P2=0.31
Age at onset, years	57.13 ± 10.12^a^	59.67 ± 8.79^a^	58.25 ± 9.67^a^	56.76 ± 8.64^a^	60.39 ± 1.01^a^	P1=0.58,P2=0.67
Site of onset, n (%)						
	U, 15 (36.6)	U, 6 (33.3)	U, 8 (47.1)	U, 4 (33.3)	U, 48 (53.3)	P1=0.37,P2=0.62
	L, 12 (29.3)	L, 7 (38.9)	L, 4 (23.5)	L, 2 (16.7)	L, 27 (30.0)	
	B, 14 (34.1)	B, 5 (27.8)	B, 5 (29.4)	B, 6 (50.0)	B, 15 (16.7)	
Family history, n	0	0	0	0	0	NA
Disease duration, months	12.17 (14.15)^b^	10.22 (8.41)^b^	8.90 (10.22)^b^	15.42 (21.62)^b^	12.52 (15.33)^b^	P1=0.09,P2=0.08
Diagnosis delay, months	11.20 (10.13)^b^	10.12 (7.92)^b^	8.10 (9.62)^b^	14.22 (18.03)^b^	12.17 (11.71)^b^	P1=0.09,P2=0.18
ALSFRS-R	41.00 (7.50)^b^	42.50 (8.50)^b^	39.00 (8.50)^b^	40.00 (8.25)^b^	39.00 (8.25)^b^	P1=0.10,P2=0.90
Progression rate						
Baseline	0.63 (0.88)^b^	0.58 (1.00)^b^	0.92 (1.33)^b^	0.49 (0.92)^b^	0.71 (0.69)^b^	P1=0.52,P2=0.16
RP/total (%)	24/41 (58.5)	10/18 (55.5)	12/17(70.6)	5/12(41.7)	56/90 (62.2)	
Follow-up	0.30 (0.34)^b^	0.31 (0.51)^b^	0.20 (0.35)^b^	0.36 (0.50)^c^	NA	P2=0.91
RP/total (%)	4/16 (25.0)	2/8 (25.0)	1/7 (14.3)	1/3(33.3)	NA	
Survival time, months	39.00 ±7.83 (23.66-54.34)^d^	23.97 ± 7.96 (8.36-39.58)^d^	30.40 ± 5.50 (19.61-41.19)^d^	36.13 (median)	34.00 ± 3.09 (27.95-40.05)^d^	P1=0.79,P2=0.23
FAB, A/T (%)	7/23 (30.4), 16.00 (3.00)^b^	3/10(30.0), 17.00 (4.00)^b^	2/8(25.0), 16.50 (3.25)^b^	2/8(25.0), 15.50 (1.75)^b^	19/59(32.2), 16.00 (4.00)^b^	P1=0.42,P2=0.31
MOCA, A/T (%)	12/22(54.5), 22.00 (5.00)^b^	5/9(55.5), 22.00 (4.50)^b^	5/8(62.5), 20.88 ± .06^a^	3/8(37.5), 22.75 ± 3.77^a^	25/52(48.1), 23.50 (8.50)^b^	P1=0.75,P2=0.56
FBI	0/20(0), 0 (3.75)^b^	0/10 (0), 0 (0)^b^	0/9(0), 0 (5.50)^b^	0/5(0), 0 (9.00)^b^	NA	P2=0.88
BDI, A/T (%)	13/26(50.0), 4.00 (8.50)^b^	4/12(33.3), 2.00 (8.75)^b^	6/12(50.0), 4.00 (9.25)^b^	5/8(62.5), 6.50 (9.75)^b^	NA	P2=0.35
HAMD, A/T (%)	15/31(48.4), 7.00 (7.00)^b^	5/14(35.7), 5.50 (6.50)^b^	5/14(35.7), 6.50 (5.50)^b^	7/10(70.0), 10.50 (11.75)^b^	NA	P2=0.06
HAMA, A/T (%)	12/31(38.7), 5.00 (8.00)^b^	3/14(57.1), 2.00 (4.50)^b^	4/14(28.6), 2.00 (8.25)^b^	7/10(70.0), 9.00 (5.00)^b^	NA	P2=0.02
PSQI, A/T (%)	1/10(10.0), 2.00 (4.00)^b^	1/6(21.4), 1.00 (3.50)^b^	1/5(20.0), 3.00 (7.00)^b^	NA	NA	NA
RBD, A/T (%)	1/10(10.0), 1.00 (2.00)^b^	1/6(16.7), 1.00 (2.00)^b^	1/5(20.0), 1.00 (3.00)^b^	NA	NA	NA
ESS, A/T (%)	0/9(0), 3.00 (4.00)^b^	0/6(0), 3.50 (3.50)^b^	0/4(0), 4.00 (4.25)^b^	NA	NA	NA

a: mean ± SD, b: median (IQR), c: median (range), d: median ± SE (95% CI), *RP* rapid progression, Control: age- and sex-matched ALS patients without *CCNF* variants and any other identified ALS-related pathogenic gene mutations. *A/T* abnormal/total, p1 value represents the comparison between pathogenic group and control group, p2 value represents the comparison between cyclin group and pest group, *IQR* interquartile range, *SD* standard deviation, *SE* standard error, *CI* confidence interval, *F* female, *M* male, *PEST* PEST sequence (short stretch of amino acids enriched in proline, glutamic acid, serine and threonine, cyclin: cyclin domains, *U* upper limbs, *L* lower limbs, *B* bulbar, *ALSFRS-R* ALS function rating scale-revised, *FAB* frontal assessment battery, *MOCA* Montreal cognitive assessment, *FBI* frontal behavioral inventory, *BDI* Beck Depression Inventory, *HAMD* Hamilton depression scale, *HAMA* Hamilton anxiety scale, *PSQI* Pittsburgh sleep quality index, *RBD* rapid eye movement sleep behavior disorder, *ESS* Epworth sleepiness scale

In addition to motor symptoms, we assessed nonmotor symptoms of patients and found that approximately 54% of patients had cognitive impairment assessed by MoCA. None of the patients met the diagnosis criteria of FTD according to the FTDC criteria [23]. Half of the patients had depressive symptoms, and 38.7% had anxiety symptoms.

Interestingly, in our ALS cohort, there were two patients (Case No. 1901 and Case No. 1120) carrying two different missense variants in *CCNF*. Case No. 1901, who carried the p.Pro487Ser and p.Arg516Gln variants of *CCNF* (predictions were 2/8 and 5/8, respectively; the latter located in the cyclin domain was considered to be a PP missense variant), developed dysarthria at the age of 65 with a disease progression rate of 1.17 and died of nutritional problems 22.5 months after onset. Another patient, Case No. 1120, carried the *CCNF* p.Ala131Ser and p.Arg751Cys variants (predictions were 6/8 and 3/8, respectively, and the former was considered to be a PP missense variant). This patient presented with dysarthria at the age of 64 with a disease progression rate of 0.26 and was lost to follow-up at 11 months of the disease course. A novel frameshift variant (p.Leu553fs) was also identified in our cohort in a patient with bulbar onset at the age of 41, with baseline and follow-up disease progression rates of 0.34 and 0.20, respectively. The survival time was more than 117 months.

In addition, 13 patients in our cohort were carriers of additional ALS-related gene variants in addition to *CCNF* variants. The clinical characteristics of the 13 individual patients are shown in Table 2. There were no significant differences in the clinical features of the 13 ALS patients who carried additional ALS-related gene variants compared with those who did not carry the CCNF gene variant and other identified pathogenic gene mutations (Supplementary Table 5). Case No. 3457 carried the *CCNF* p.Arg516Trp variant (which was considered PP) and *ALS2* p.Pro955Arg variant (which was considered PP), neither of which has been reported previously. This patient had extremely rapid disease progression with a progression rate of 2.32 and died from choking approximately 16 months after disease onset. Two female patients (Case Nos. 1149 and 1168) in our cohort carried additional variants besides *CCNF* p.Gly716Ser variant which was predicted to be benign. Case No. 1168 was a carrier of an additional *TIA1* variant (p.Phe214Tyr, prediction 2/6) and present with a fast progression rate of 0.66 at baseline. This patient survived for more than 152 months and was still alive at the last follow-up. Case No. 1149 was a carrier of an additional *CHMP2B* variant (p.Asp151Glu, prediction 4/8) but was lost to follow-up.

**Table 2:** Clinical features of *CCNF* variant carriers who had other ALS-related gene variants

Case No.	*CCNF* variant	Age at onset, y	Disease duration, m	Sex	Site of onset	ALSFRS-R	Progression rate	Survival time, m	Outcome	Other gene variant
1046	p.Ser222Pro	56.49	12.17	M	L	46	0.16	12.17	2	*FIG4(p.Pro851Leu)*
1149	p.Gly716Ser	43.44	35.70	F	B	45	0.08	35.70	2	*CHMP2B(p.Asp151Glu)*
1168	p.Gly716Ser	46.97	42.37	F	B	20	0.66	152.13	1	*TIA1(p.Phe214Tyr)*
1228	p.Arg340Gln	44.49	8.90	M	B	36	1.35	8.90	2	*FIG4(c.1389-2A>G)*
1363	p.Pro487Ser	69.81	11.60	M	B	39	0.78	11.60	2	*EWSR1(p.Gly589Ser)*
2330	p.Ala446Thr	45.26	6.30	M	B	44	0.63	39.00	0	*SETX(p.Asn1654Ser)*
3017	p.Arg568Trp	74.25	5.47	M	B	41	1.28	14.90	0	*CHMP2B(p.Lys8Asn),ALS2(p.Asp298Tyr)*
3199	p.Thr511Asn	69.55	18.30	F	U	37	0.60	23.97	0	*PNPLA6(p.Ala344Thr)*
3327	p.Pro550Leu	44.44	22.20	M	U	41	0.32	59.77	1	*DAO(p.Gly129Ser)*
3355	p.Pro27Ser	71.11	23.53	F	L	43	0.21	59.33	0	*SETX(p.Thr1188Asn)*
3457	p.Arg516Trp	61.18	6.03	F	L	34	2.32	15.90	0	*ALS2(p.Pro955Arg)*
3474	p.Pro487Ser	71.20	5.53	M	U	36	2.17	32.47	0	*FIG4(p.Thr270Ile)*
3493	p.Arg344Lys	63.44	14.93	M	U	41	0.47	15.67	0	*NEFH(p.Glu700Ala)*

*y* years, *m* months, 0: death, 1: alive, 2: loss, *M* male, *F* female, *U* upper limbs, *L* lower limbs, *B* bulbar

### Literature Overview

**Fig. 3:**
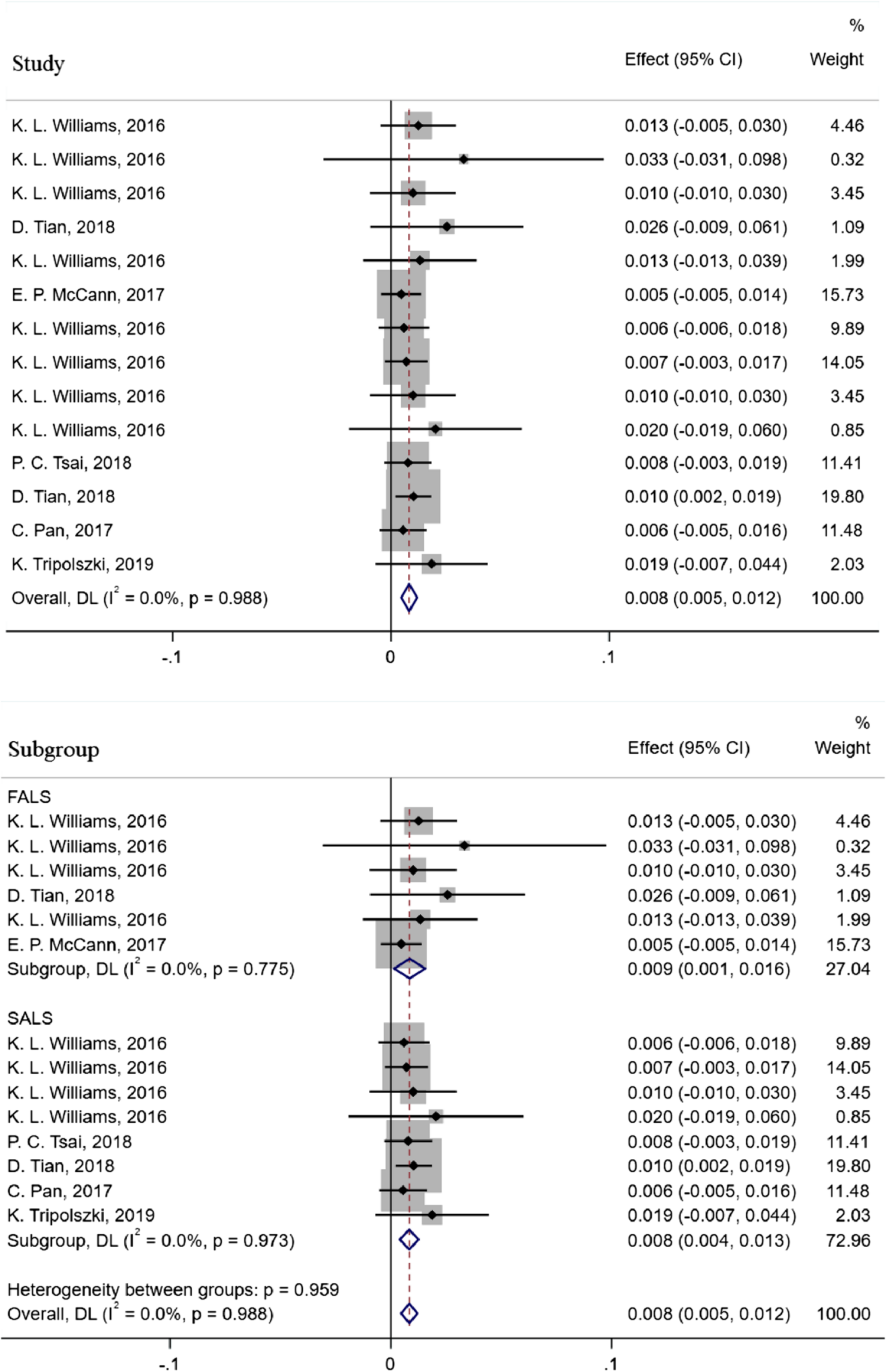
The frequency of *CCNF* variants

After systematically reviewing the literature, seven relevant studies consisting of 59 ALS/FTD patients with *CCNF* variants, including 15 Asian patients [17–19] and 34 Caucasian patients [5, 20–22], were analyzed. A total of 43 *CCNF* variants in ALS/FTD patients were reported [5, 17–22], including one stop-gain variant, one frameshift variant, and 41 missense variants (Supplementary Table 4). There were no significant differences in the frequency of *CCNF* variants in FALS and SALS patients between Caucasian and Asian populations (1.9% (2/107) vs. 2.6% (2/78), *p*=0.26; 1.0% (6/575) vs. 0.8% (14/1616), *p*=0.30). The overall variant frequency was approximately 0.8% (95% CI 0.5–1.2%), with 0.9% in the FALS and 0.8% in the SALS (Fig. 3). In addition, one FTD patient carrying the p.S621G variant of *CCNF* (1.3%, 1/75) was identified in a FALS cohort (Supplementary Table 4) [5].

The mean age of onset of previously reported *CCNF* carriers was 52.62 ± 13.59 years, the proportion of bulbar onset accounted for 10%, and the median survival time was approximately 43.00 ± 6.12 months. There were no statistically significant differences in the age of onset and site of onset between Caucasian and Asian *CCNF* carriers. The ratio of males to females was significantly higher in Asian populations than in Caucasian populations (10/1 vs. 4/7, *p*=0.02) (Table 3).

**Table 3:** Previously reported clinical features of ALS and/or FTD patients with *CCNF* gene variants

	All (*n*=22)	Asians (*n*=11)	Caucasians (*n*=11)	*p* value
Mutation frequency	0.8%	0.8%	0.9%	0.62
	FALS:0.9%, SALS: 0.8%	FALS: 2.6% (2/78), SALS: 0.8% (14/1616)	FALS: 1.0% (6/575), SALS: 1.9% (2/107)	0.26,0.30
Sex, F/M	8/14	1/10	7/4	0.02
Age at onset, years, n	21, 52.62 ±13.59^a^	11, 54.07 ± 6.52^a^	10, 51.03 ± 18.91^a^	0.61
Site of onset, n (%)				
	S, 17 (89.5)	S, 11 (100.0)	S, 6(75.0)	0.16
	B, 2 (10.5)	B, 0(0)	B, 2(25.0)	
Cognition, A/T (%)	2/20 (10.0)	0/9(0)	2/11(18.2)	0.25
Survival time, months, n	13	11	2	
Median ± SE (95% CI)	43.00 ± 6.12 (31.01 – 54.99)^d^	43.00 ± 5.91 (31.41 – 54.99)^d^	24.00 (median)	NA

a: mean ± SD, *A/T* abnormal/total, *F* female, *M* male, d: median ± SE (95% CI), *SD* standard deviation, *SE* standard error, *CI* confidence interval, *U* upper limbs, *L* lower limbs, *B* bulbar, *p* value represents the comparison between LoF group and missense variant group

Based on the protein level, seven variants in 16 reported ALS patients were located in the PEST sequence, 13 variants in 16 reported ALS patients were located in the cyclin domains, and five variants in five patients were located in the F-box domain (Fig. 1B). Due to insufficient clinical data, it was not possible to compare whether there were differences in the clinical characteristics among ALS patients with variants in the three domains of CCNF.

## Discussion

We investigated the genetic spectrum and clinical phenotype of *CCNF* variants through our large Chinese ALS cohort and literature review. First, the PP variant frequency of *CCNF* in our cohort (1.1%) was comparable to that in previous studies (0.8%). Second, the 11 novel PP missense variants identified in our cohort expand the genetic variant spectrum of *CCNF*. Third, our study found that nearly one-third of *CCNF* variant carriers had additional variants in other ALS-related genes in addition to *CCNF* variants. Finally, more than half of *CCNF* carriers in our cohort had cognitive impairment, but no patients met the diagnosis criteria of FTD.

The *CCNF* gene encodes cyclin F, which is a founding member of the F-box protein family that mainly includes three functional modules: a pseudocatalytic module containing the F-box domain and NLS1, a substrate complement module containing two cyclin domains, and a regulatory module containing NLS2 and PEST (a short amino acid rich in proline, glutamic acid, serine and threonine) [24]. Cyclin F is essential for genome stability and regulates deoxyribonucleotide triphosphate levels, centrosome duplication and spindle formation by targeting and regulating the levels of SCF^cyclin F^ substrates [25, 26]. Recently, it was also found that the expression of *CCNF* in the motor neuron-like cell line NSC-34 changes after exposure to oxidative stress [27]. CCNF can also affect the activity of valosin-containing protein (VCP) in the cytoplasm [28].

In our cohort, a total of 41 ALS patients were carriers of 29 variants in the *CCNF* gene, but only 15 missense variants were identified as deleterious by software analyses. Therefore, the variant frequency of *CCNF* in our ALS cohort was 1.1% (15/1587), which was similar to the results of previous studies [5, 17–20]. A previous study found an enrichment for rare protein alterations in *CCNF* (MAF < 0.0001) among SALS patients compared to control individuals [5]. However, this study also identified a *CCNF* frameshift variant (p.L372fs) that did not segregate with disease, suggesting that a dominant gain in toxic function may be required for the pathogenicity of mutant *CCNF* [5]. One patient with the frameshift variant (p.Leu553fs) was also found in our cohort. However, because of the lack of genetic information on relatives, it was not possible to determine whether the variant segregated with the disease. Therefore, whether the functional consequences of *CCNF* variants lead to increased functional toxicity or loss of dominant negative function or haploinsufficiency remains to be determined by more studies.

Currently, studies have not found a clear relationship between the location of *CCNF* variants and the clinical phenotype. Two variants (p.Pro27Ser and p.Arg21Gln) were located in the NLS1 domain, which mediates interactions with other components of the SCF ubiquitin-protein ligase complex to achieve ubiquitination of target substrates [24]. This is the first report of a variant in the NLS1 domain. Both variants were considered deleterious by software (predictions of 4/8 and 5/8, respectively). In our cohort, there was also a newly discovered variant (p.Arg568Trp) located in the NLS2 domain, which was considered to be harmful with a prediction of 4/8. The patient carrying this variant had extremely rapid disease progression, with a progression rate of 1.28, and died of sputum asphyxia less than 15 months after onset. The p.Arg568Trp variant has not been reported previously. However, two variants (p.R574Q and p.R572W) located in the NLS2 domain have been reported in European populations and are also considered to be pathogenic variants [5, 20].

Ten variants were located in the cyclin domains in our cohort, eight of which (all except the p.Pro487Ser and p.Arg344Lys variants) were predicted to be PP (prediction ≥ 4/8). Interestingly, the benign *CCNF* p.Pro487Ser variant (prediction 2/8) was present in six patients in our ALS cohort, and this variant was previously reported in an Asian patient [17]. In our cohort, a benign p. Arg344Lys variant (prediction 2/8) identified in two patients has been previously reported once in both European and Asian populations [5, 19].

Seven variants located in the PEST sequence were all predicted to be benign by software analyses (prediction<4/8) in our study, and none have been reported previously. However, the p.S621G variant located in the PEST sequence has been reported repeatedly in Caucasian FALS/FTD patients [5, 22]. The p.S621G variant prevents the phosphorylation of casein kinase II (CK2) and increases the lys48-ubiquitin activity, resulting in dysfunction of the autophagy degradation pathway [5, 29, 30]. It was also found that zebrafish with the p.S621G variant had disrupted axonal growth, suggesting a toxic gain-of-function mechanism in the pathogenesis of ALS [31]. The p.S621G variant was not found in our cohort, and all variants identified in our cohort were not located at the identified phosphorylation sites [29]. Therefore, based on the results predicted by software and the current literature review, we considered these seven variants located in the PEST sequence in our cohort to be likely benign, but more basic experimental studies are needed to explore the pathogenicity and pathogenesis of *CCNF* variants located in the PEST sequence.

Four *CCNF* variants (p.Gly161Arg, p.Ser222Pro, p.Ala131Ser, and p.Arg123Cys) predicted to be PP in our study were not located in any functional domain. Among them, the p.Gly161Arg variant of *CCNF* has been reported previously in both Caucasian and Asian populations. In the Asian populations, the p.Gly161Arg variant was found in a 43-year-old man who presented with rapidly progressive left-hand weakness and died of respiratory failure two years after onset [5, 20]. In our cohort, the patient carrying the p.Gly161Arg variant also had a rapid progression rate of 1.5, but the survival time of this patient could not be obtained due to loss of follow-up. A previously reported Asian carrier of the p.Ser222Pro variant of *CCNF* had a relatively long survival time of more than 60 months [17]. The in vitro functional study found that the p.Ser222Pro variant of *CCNF* had a deleterious effect on cyclin F-mediated proteasome degradation, and UPS damage may occur upstream of the proteasome through abnormal ubiquitination or transport to the proteasome [5, 17]. The p.Ala131Ser and p.Arg123Cys variants of *CCNF* have not been reported previously. Although they are not located in any functional domain, they may also be related to the folding and aggregation of abnormal proteins. Further basic studies are needed to explore the structural or functional changes of these variants.

Currently, this study is the largest sample size of *CCNF* variant carriers and clinical characteristics analysis. The frequency of PP variants in *CCNF* in our study was similar to that in previous studies, but there were differences in the results of sex ratio, site of onset, survival, and cognitive function. The *CCNF* carriers in our ALS cohort had a late age of onset (59.67 years vs. 52.62 years) and a large proportion of bulbar onset (27.8 vs. 10.5%), especially among Asian patients, where bulbar onset has not been reported before. In addition, patients with PP variants of *CCNF* in our study had a shorter median survival than previously reported results (24.0 months vs. 43.0 months). The proportion of male patients was similar between our cohort and Caucasian patients. In addition to the ALS phenotype, a previous study on FALS and FTD patient cohorts found *CCNF* carriers with primary lateral sclerosis [5], which was not found in our cohort. This may be because the current cohort only enrolled ALS patients. No significant differences in clinical manifestations were found in patients carrying *CCNF* variants in different domains in our cohort, but *CCNF* variant carriers with variants located in cyclin domains tended to have a faster disease progression rate than carriers with variants located in PEST domains (0.92 vs. 0.49). However, the difference was not statistically significant. This was consistent with our finding that all variants located in the PEST sequence were likely benign, while the majority of variants located in the cyclin domains were probably pathogenic.

Our study has the following limitations. First, we did not have genetic information from the relatives of carriers of *CCNF* variants, so we cannot estimate whether these variants segregate with disease. Second, there are few studies on *CCNF* variants in ALS, and the correlation between *CCNF* carriers and clinical phenotypes cannot be clarified. Third, we assessed the pathogenicity of missense variants only by in silico tools, without further basic experiments, which could lead to misjudgments of pathogenicity.

## Conclusion

In conclusion, we screened Chinese ALS patients for *CCNF* variants and found that such variants were common, especially in SALS patients. Our study expands the *CCNF* genetic variant spectrum in ALS patients; however, the pathogenic mechanisms and pathogenicity of many novel variants are poorly understood. There are differences in clinical characteristics between ALS patients in our cohort and those of previous studies. More follow-up observations and basic studies are needed to elucidate the pathogenic mechanisms and genotype-phenotype associations of *CCNF* variants.

## Supplementary Information

ESM 1 Supplementary Table 1 Information on *CCNF* variants in ALS patients. Supplementary Table 2 Protein function, structure and nucleic acid conservation prediction of *CCNF* variants in ALS. Supplementary Table 3 Information on additional ALS-related gene variants in ALS patients with *CCNF* variants. Supplementary Table 4 Gene analysis and clinical characteristics of *CCNF* variants in previously reported ALS patients. Supplementary table 5 Clinical features of *CCNF* variants carriers who had other ALS-related gene variants. Supplementary table 6 The ALS-related genes in the study (XLSX 41 kb)

## Data Availability

Anonymized data will be shared upon request with any qualified investigator.
